# pH-Dependent Effects of Short-chain Carboxylic Acids and Buffer Systems On *Clostridioides difficile *in Vitro and in Vivo

**DOI:** 10.1007/s00248-026-02694-6

**Published:** 2026-01-14

**Authors:** Lucía Huertas-Díaz, Jiri Hosek, Ditte Gram-Hansen, Remo Frei, Caroline Roduit, Mari Sasaki, Roger P. Lauener, Clarissa Schwab, C. K.-CARE Study Group, Thomas Bieber, Peter Schmid-Grendelmeier, Cezmi A. Akdis, Marie-Charlotte Brüggen, Claudio Rhyner

**Affiliations:** 1 Department of Biological and Chemical Engineering, Aarhus University, Gustav Wieds Vej 10, 8000, Aarhus C, Denmark; 2 Christine Kühne-Center for Allergy Research and Education (CK-CARE), Davos, Switzerland; 3 Department of Biomedical Research, University of Bern, Bern, Switzerland; 4 Division of Paediatric Respiratory Medicine and Allergology, Department of Paediatrics, Inselspital, Bern University Hospital, University of Bern, Bern, Switzerland; 5 Children’s Hospital St., Gallen, St. Gallen, Switzerland

**Keywords:** Clostridoides difficile, Short-chain carboyxlic acids, pH, Buffer

## Abstract

The spore-forming anaerobe *Clostridioides difficile* colonizes the highly dynamic gut environment early after birth, frequently without causing disease. In this study, we aimed to determine how environmental conditions indicative of the infant gut impacted prevalence and physiology of *C. difficile*. We examined the effect of pH, fermentation derived short-chain carboxylic acids (SCCA) and buffering systems combining in vitro and in vivo analysis, and experimental and modelling approaches. In vivo, the prevalence of *Clostridioides* significantly increased between 3 months (30.2%) and 12 months (56.2%) after birth. At 12 months, the occurrence of *Clostridioides* was the highest in feces with near neutral pH (6.7 (IQR 6.5‒7.3). In vitro, *C. difficile* showed pH-dependent growth and metabolic activity with an optimum around pH 5.8–6.3. Most SCCA conferred antimicrobial activity at pH 5.2 and 6.1 while at pH 6.5, high concentrations of SCCA promoted growth. The presence of phosphate buffer enhanced antimicrobial activity of SCCA, particularly at lower pH values (5.2–5.8). Two multilinear regression models indicated that ionic strength was inversely related to optical density in vitro, while in vivo*,* the abundance of *Clostridioides* was inversely linked to the presence of undissociated SCCA. Together, this study highlights that the that occurrence and performance of the opportunistic pathogen *C. difficile* was affected by chemical systems such as pH, the presence of buffer systems and concentration and chemical state of SCCA. Our results suggest novel targets that could be modulated to impact *C. difficile* colonization.

**Supplementary Information:**

The online version contains supplementary material available at 10.1007/s00248-026-02694-6.

## Background

*Clostridioides difficile* is an anaerobic, spore-forming opportunistic pathogen that causes* C. difficile* infection (CDI) frequently after antibiotic treatment [1, 2]. *C. difficile* is present in the feces of up to 10% of healthy adults [3] and is metabolically versatile [4, 5]. Strains of *C. difficile* can utilize a broad range of substrates, including monosaccharides, amino acids, CO_2_ through the Wood–Ljungdahl pathway, and the short-chain carboxylic acid (SCCA) succinate [6]. Previous studies demonstrated that *C. difficile* colonizes the infant gut early after birth [7], often without causing any disease. The probability of colonization by *C. difficile* differed between exclusively breastfed infants (> 25%) compared to infants that received formula (~ 50%) [8]. In the Swiss birth cohort ‘Childhood AlleRgy nutrition and Environment’ (CARE), occurrence and abundance of *Peptostreptococcaceae*, which includes *C. difficile*, increased from three to six and 12 months [9, 10] indicating a distinct colonization pattern linked to infant gut microbiota development.

During the first year of life, composition of the gut microbiota is highly dynamic [10, 11]. The microbiota of breastfed infants is mainly dominated by *Bifidobacterium* spp. [11], which degrade and ferment human milk oligosaccharides [12] composed of galactose, glucose, N-acetylglucosamine, sialic acid and fucose to acetate, formate and lactate [13]. With the introduction of solid food, the microbiota becomes more diverse and is mainly composed of *Lachnospiraceae, Ruminococcaceae* and *Bacteroidaceae.* Fermentation capacity increases [11] and acetate, butyrate and propionate are the major metabolites present in fecal samples of infants at one year of age [11]. The fermentation intermediates lactate, formate and succinate are mainly used for microbial cross-feeding [14] and are generally recovered infrequently and at lower levels [11]. Other SCCA such as valerate that is produced through chain elongation or via amino acid fermentation through the Stickland reaction [4, 15], are frequently detected in feces of infants [16, 17] and adults [18]. The branched SCCA, isovalerate and isobutyrate, are formed by Stickland reaction from amino acids [4].

Beyond their role in microbial cross-feeding [15], SCCA confer antimicrobial activity [15, 19]. As weak acids with a carboxyl group, SCCA can modify environmental conditions through acidification. In vivo, phosphate and bicarbonate buffer systems maintain the pH of the environment [20, 21]. Such systems provide acids and/or bases to buffer in the presence of H + or OH-^22^. In addition, SCCA can directly interact with microbial cells [22]. Based on the weak acid theory, antimicrobial activity of SCCA depends on the pK_a_ and the environmental pH [22], as mainly the undissociated SCCA penetrate the bacterial membrane to dissociate intracellularly [19, 22]. Additionally, compound hydrophobicity defined by the octanol/water partition coefficient (log *K*_ow_) impacts antimicrobial efficiency as polar compounds can cross the lipid membrane barrier more easily [23]. Among the SCCA that are common in the gut ecosystem, the pK_a_ and log *K*_*ow*_ ranges from 3.75―5.61 and −0.72―1.39 (Table 1). With the exception of succinate, the major SCCA present in the infant gut possess one carboxyl group (Table 1).

**Table 1:** Chemical properties from SCCA. Hydrophobicity as octanol/water partition coefficient (Log *K*_ow_). Dissociation strength (pK_a_)

SCCA	Carbon number in backbone	Additional functional groups	pK_a_	Log *K*_ow_
Acetate	2	-	4.76	−0.17
Propionate	3	-	4.89	0.33
Butyrate	4	-	4.82	0.79
Formate	1	-	3.75	−0.54
Lactate	3	―OH	3.85	−0.72
Valerate	5	-	4.82	1.39
Succinate	4	―COOH	pKa_1_ 4.16; pKa_2_ 5.61	−0.59

Gut microbes are consistently exposed to a variety of SCCA, and SCCA composition is especially dynamic during the first year of life [11, 24]. There is little knowledge on the interdependence of antimicrobial activity related to the chemical state of SCCA, community composition and/or the presence of specific microbial taxa. While *C. difficile* often remains asymptomatic in infants, this study on its occurrence related to environmental parameters such as pH and SCCA can lead to mitigate strategies to counteract adverse effects of *C. difficile*, possibly also later in life. To shed light on possible interactions of *C. difficile* and fermentation-derived SCCA that occur in the infant gut, we combined in vivo analysis of fecal samples from the CARE study with in vitro growth studies focusing on *C. difficile* and SCCA interactions at different pH and in the presence of different buffering systems.

## Materials and Methods

### Reanalysis of 16S rRNA Gene Libraries and SCCA Profiles, and Fecal pH Measurements

Fecal samples were collected from a sub-group of the CARE birth cohort, an ongoing study from St.Gallen Switzerland, which aims to find environmental exposures in the first year of life, that influence the development of the microbiome early in life. The fecal samples used in this study were obtained from *n* = *69* infants and collected during exclusive breastfeeding (3–4 months of age), during and after the introduction of solid foods (6–7 months) and at around one year of age (12–13 months). To re-analyze microbiota composition, we retrieved the 16S rRNA gene dataset generated by Appert et al*.* [9] from ENA (PRJNA616703) and processed the data as described [25]. Briefly, primer sequences were removed using Cutadapt (v. 4.4; -O 12 –discard-untrimmed -g GTGCCAGCMGCCGCGGTAA –G GGACTACHVGGGTWTCTAAT –pair-adapters –minimum-length 75) (22) and only inserts that contained both primers and were at least 75 bases were kept for downstream analysis. Reads were quality filtered using the filterAndTrim function of the dada2 package (maxEE = 2, truncQ = 3, minLen = 150, trimRight = 40, trimLeft = 40). The learnErrors and dada functions were used to calculate sample inference using pool = pseudo as a parameter. Reads were merged using the mergePairs function and chimeras were removed with removeBimeraDenovo (method = pooled). The remaining amplicon sequence variants were taxonomically annotated using the IDTAXA classifier [26] in combination with the Silva v. 138 database [27]. We combined taxonomic classification based on 16S rRNA gene amplicon sequencing with total bacteria counts determined with quantitative PCR [9] for quantitative microbiota profiling of *Clostridioides* abundance.

SCCA data was obtained from Appert et al*.* [9] and Sasaki et al*.* [28]. pH was recorded for this study from fecal water from 4 and 35 samples collected at 6 and 12 months, respectively. Fecal pH in samples collected at 12 months were stratified in quartiles based on no/low, medium and high cell counts. The median detection limit was estimated based on the minimal number of one read/sample as 5.3 log_10_ cells/g feces.

### Strains, Culture Media and Growth Conditions

All media ingredients were purchased from Merck, unless otherwise stated. *Clostridioides difficile* DSMZ 12056 was purchased from the German Collection of Microorganisms and Cell Cultures (DSMZ) and was stored at −80 °C as glycerol stock (30% glycerol). *C. difficile* was routinely cultivated in Wilkins-Chalgren broth supplemented with 5 g/L soya peptone (Biolife), 1 g/L Tween 80, 0.5 g/L L-cysteine-HCl (WCSP) and agar 15 g/L (VWR). All components were solubilized in milliQ water. The pH was adjusted to 7.2, media were boiled, and L-cysteine-HCl was added after cooling down and before the start of degassing with CO_2_. All media were autoclaved 121 °C for 15 min before use [10]. To activate frozen stock cultures, *C. difficile* was grown on WCSP agar plates in the anaerobic chamber (10% CO_2_, 5% H_2_, 85% N_2_, Baker Ruskinn) for three days. Individual colonies were picked and inoculated into 10 mL WCSP broth. Cultures were subcultured twice (2% inoculum) in WCSP to obtain working cultures.

We determined the impact of pH, the presence of phosphate buffer (PB) and/or SCCA on growth and metabolic activity in Hungate tubes. The pH of WSCP was adjusted prior to autoclaving to + 1 pH unit above the targeted pH with NaOH or HCl to obtain the desired pH after autoclaving. To test the impact of a buffering system, Na_2_HPO_4_ and NaH_2_PO_4_ · H_2_O were added (10, 50 and 100 mM) to achieve a final pH of 5.2, 5.8, 6.1 and 6.5. The evaluated pH were selected according to the observed pH *in vivo* [29]. SCCA were added at concentrations of ranging from 60‒160 mM (Suppl. Table S1), to cover a wide range of concentrations reported in the infant feces [10]. Working cultures were added at 2% (vol/vol). After 24 h of incubation at 37 °C, optical density was recorded with a McFarland Densitometer (Grant-Bio). Samples (1 mL) were collected at t = 0 and 24 h, centrifuged, and supernatants were kept at −20 °C until further analysis. Each experiment was run in biological triplicates unless otherwise indicated, results shown are average replicates with standard deviation.

### Determination of Antimicrobial Activity in 96-Well Microtiter Plates

The antimicrobial activity of SCCA was tested using two-fold broth dilution broth assays [30] (Suppl. Fig. S1). To prepare stock solutions, SCCA were added to WCSP medium. Final concentrations were determined with high-performance liquid chromatography (HPLC-RI) as outlined below. The pH was adjusted; stock solutions were filtered (0.22 µm) and kept for 24 h inside the anaerobic chamber prior to experiments. The 96-well microtiter plates were filled with 100 μL anaerobically prepared WCSP with pH 5.2, 6.1, or 6.5 except column 2. SCCA stocks were added (200 μL) to column 2, and samples were serially diluted (100 μL, two-fold dilution). Working cultures (1 mL) were added to 9 mL of WCSP and supplied to the wells at 10% (vol/vol). Plates were incubated in an anaerobic bench at 37 °C for 24 h. Positive (cultivation medium without SCCA) and negative controls (sterile media instead of cell suspension), were included in each plate. Growth was evaluated by measuring the cell density at 600 nm (OD_600nm_) using the Infinite M200 Pro Plate Reader (TECAN). To determine the minimum inhibitory concentration to reduce cell density to 50% (MIC_50_), we fitted the OD_600_ values into four parameter logistic regression curve [23]; the inflection point of the resulting curve represented the MIC_50_ value. Each experiment was run in biological triplicates unless otherwise indicated, results shown are average of replicates with standard deviation.

### Glucose and SCCA Analysis by HPLC-RI and Calculation of Ionic Strength and Dissociation State

In addition to the analysis of optical density as an indicator of growth, we determined glucose consumption and levels of SCCA to test for metabolic activity. Glucose and SCCA levels were determined in supernatants collected from Hungate tubes at 0 and 24 h of incubation. A 1260 Infinity II LC System equipped with a Hi-Plex H guard (7.7 × 50 mm, 8 µm) and separation (300 × 7.7 mm) columns and a refractive index detector (all Agilent) was used. All samples were diluted 1:1 with HPLC elution buffer (5 mM H_2_SO_4_). The samples (10 μL injection volume) were eluted 5 mM H_2_SO_4_ at a flow rate of 0.6 mL/min at 40 °C. Metabolites were quantified using external standards and Chromeleon Console (v. 7.7.2.10) for analysis. Retention time for the metabolites were 11.5 min for glucose, 15.4 min for succinate, 15.7 min for lactate, 16.9 min for formate, 18.7 min for acetate, 22.3 min for propionate, 24.8 min for ethanol, 26.2 min for isobutyrate, 28.4 min for butyrate, and 34.2 min for isovalerate, and 40.9 min for valerate.

To estimate ionic strength and dissociation state of SCCA, we employed the software Visual MINTEQ v. 3.1 [31]. For all calculations we set the temperature to 37 °C and employed pH values recorded in blank controls or from fecal water. We used SCCA concentrations determined with HPLC-RI, and estimated PB concentrations of 10, 50, 100 mM for in vitro assays, and 20 mM as an estimation of the concentration found in the infant gut [32].

### Multilinear Regression Models

We explored the combinatory effect of PB with each SCCA on optical density using multilinear regression (MLR) models. In MLR model 1, we treated pH and PB as numerical variables and SCCA as a categorical variable that was converted in R with the function as.factor(). To fit the MLR model 1 in R v. 4.3.3, we applied the function lm() that is part of the basic package ‘stats’ [33], which uses ordinary least squares for fitting. The MLR model 1 of interaction of effects of SCCA and PB on optical density was based on Equation 1 (Eq. 1):

$$lm(Formula=Optical density \sim pH+SCCA:PB)$$

We explored more possible designs, for e.g. adding PB as a variable or using higher order polynomial of explanatory variables. To ensure that the best model was used, we tested the designs using Akaike information criterion (AIC) and selected the option with the smallest AIC value.

To investigate the relationship between environmental properties and growth we fitted a second MLR model 2 of optical density data on pH, ionic strength and presence of various types of SCCA. We treated pH and ionic strength as numerical variables and SCCA as a categorical variable. The regression was fitted with Eq. 2:

$$lm(Formula=Optical density \sim pH+ ionic strength+SCCA)$$

### General Statistics

Statistical analyses were performed using R Studio (v. R-4.4.1). Normality was tested by Shapiro-test, and when data was not normally distributed, non-parametric tests were employed. We employed one ANOVA for optical density and substrate utilization and metabolite formation with Tukey-adjusted pairwise comparisons. Non-parametric analyses were employed to calculate significance using Kruskal–Wallis for pairwise comparison and Dunn’s test for multiple comparison adjusted by FDR. Chi square analysis was performed with the package ‘rstatix’ [34]. For the Factor Analysis of Mixed Data (FAMD) we employed the package ‘FactoMineR’ [35] and included *n* = *35* samples collected at 1 year with recorded pH values. Qualitative pH categories were classified according to the quartiles; Low pH (pH ≤ 5.3, *n* = *9,* Q1); Medium pH (5.3 ≤ pH ≤ 6.5, *n* = *16,* Q2) and High pH (pH ≥ 6.5, *n* = *10,* Q3). The presence of *C. difficile* was qualitatively classified in samples with detectable *C. difficile* (*n* = *17*, > 4.9 log_10_ cells/g feces) or non-detectable *C. difficile* (*n* = *18*).

## Results and Discussion

### *Clostridioides* Abundance and Occurrence Related to pH and Presence of SCCA In Vivo

**Fig. 1:**
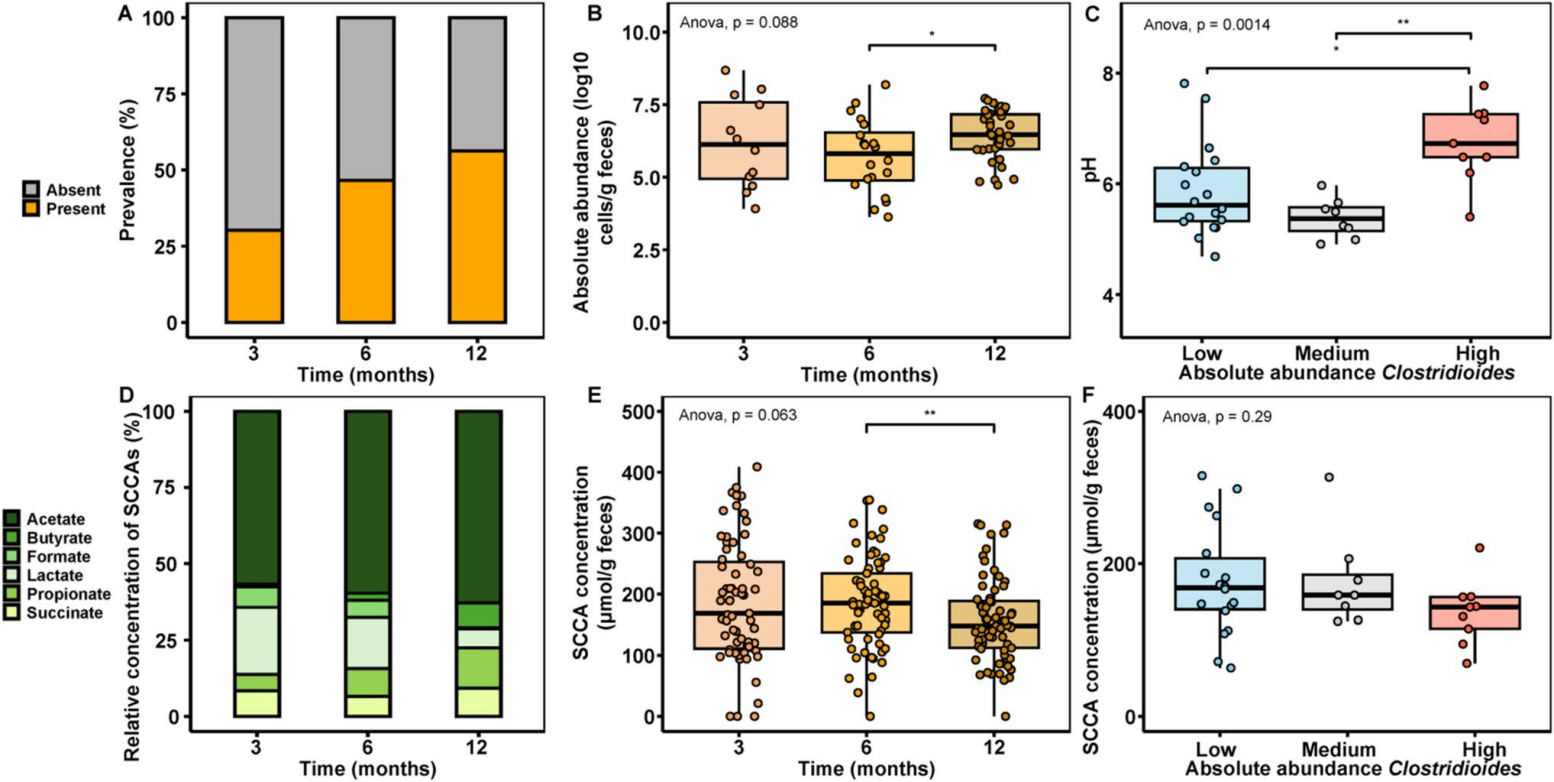
Occurrence of *Clostridoides* and levels of short-chain carboxylic acids (SCCA) in fecal samples. Fecal samples (*n*=165 samples in total) were obtained from the CARE cohort collected at 3, 6 and 12 months of life, and prevalence and abundance of *Clostridoides* was determined using quantitative microbiota profiling. pH was measured from fecal water of *n*=35 samples collected at 12 months, and fermentation metabolites were quantified using HPLC-RI. (**A**) Prevalence (%) of Clostridioides at 3, 6 and 12 months. (**B**) Absolute abundance of *Clostridoides* at 3, 6 and 12 months. (**C**) Fecal pH in samples at 12 months were stratified depending on *Clostridoides* abundance in quartiles (now/low, medium and high cell counts). (**D**) Proportion (%) of SCCA at 3, 6 and 12 months. (**E**) Total SCCA concentrations (micromol/g feces) at 3, 6 and 12 months. (**F**) Total SCCA concentrations in feces with different Clostridoides abundance classified in quartiles based on no/low, medium and high cell counts. Statisical tests were conducted using Wilcox test and ANOVA, a *p*<0.05 was considered significant

We evaluated the relationship between fecal SCCA levels, pH and abundance of the *Clostridioides* genus by re-analysis of the data generated by Appert et al*.* [9] of the CARE birth cohort study. The median number of analysed reads per processed sample was 31,228 (range 3,539–118,418 reads). Based on quantitative microbiota profiling, the genus *Clostridioides* was detected in 30.2% (16/53) of infants at the age of 3 months, and the proportion of infants carrying *Clostridioides* was higher at 6 (46.6%, 27/58) and 12 months (56.2%, 36/64) (Chi square, *p* = *0.001*) (Fig. 1A). The median abundance for carriers of *Clostridioides* was 6.1 log_10_ cells/g feces (interquartile range (IQR) 4.9‒7.6) at 3 months, 5.8 log_10_ cells/g feces (IQR 4.9‒6.5) at 6 months and increased (*p* < *0.05*) at 12 months to 6.5 log_10_ cells/g feces (IQR 6.0‒7.2) (Fig. 1B). An increase in prevalence of *Clostridioides* was also observed in other cohorts from 19% at 2 months (116/624), to 37% at 6 (221/606), and 40% at 12 months (227/574) [36].

In addition, we determined fecal pH of *n* = *35* samples collected at 12–13 months, and *n* = *4* samples at 6 months. The median pH at 6 months was 4.44 while the median pH was 5.68 in samples collected at 12–13 months, in line with previous observations [37]. One limitation of our data was the small sample size at 6 months due to a limited amount of fecal sample. To relate *Clostridioides* abundance with fecal pH, we stratified infants based on fecal cell counts in the following quartiles: First quartile (Q1), ‘no detection/low *Clostridioides* abundance' < 4.9 log_10_ cells/g feces *n* = *17*), Q2, ‘medium’ *Clostridioides* abundance, 4.9 log_10_ cells/g feces < *Clostridioides* < 6.4 log_10_ cells/g feces, *n* = *8*) and Q3, ‘high’ *Clostridioides* abundance (Q3, ≥ 6.4 log_10_ cells/g feces, *n* = *9*). For the ‘high’ group, the median pH (6.7 (IQR 6.5‒7.3)) was higher (*p* < *0.05*) compared to ‘medium’ 5.4 (IQR 5.1‒5.6) and ‘no’ (5.6 (IQR 5.3‒6.3)) (Fig. 1C). Also in other studies, *C. difficile* was rarely detected if the fecal pH was below 6.0 [38]; and 96% of patients with stool pH below 7.0 tested negative for *C. difficile* infection [39]. As we observed in this study, *C. difficile* can also contribute to SCCA formation and thereby acidification of the gut environment. Nonetheless, with a median abundance of 6.5 log_10_ cells/g feces, the abundance of *Clostridioides* within the fecal microbiota was rather low.

As fecal pH might relate to presence and/or distribution of SCCA, we re-evaluated SCCA profiles at 3, 6 and 12 months. Acetate was the predominant SCCA contributing approx. 50% to all SCCA at all three time points. Lactate (21.9%) and succinate (8.4%) were the second and third most abundant metabolites at 3 months. Propionate (13.2%), succinate (9.3%) and butyrate (8.1%) were contributed a higher proportion at 12 months (Fig. 1D). Total concentration of SCCA decreased between 6 (185.1, IQR 137.8‒234.0 µmol/g feces) and 12 months (148.3, IQR 112.2‒188.6 µmol/g feces, *p* < *0.05*) (Fig. 1E), possibly due to increased cross-feeding activity and lower concentrations of fermentation intermediates lactate, succinate and formate at 12 months. At 12 months, *Clostridioides* abundance did not relate to total SCCA levels (143.1‒168.3 µmol/g feces range) (Fig. 1F).

These observations made in vivo suggest that environmental conditions characterized by high pH favored the occurrence and abundance of *Clostridioides*.

### Growth and Fermentative Activity Related to pH conditions In Vitro

To further investigate the relationship of pH and *C. difficile* growth, we conducted in vitro assays using WSCP medium in Hungate tubes and determined final optical density and fermentation metabolites after growth at pH 4.7–7.3.

Highest optical density was observed at pH 5.8 (8.5 ± 0.3 MF), pH 6.1 (7.7 ± 0.1 MF) and pH 5.5 (7.5 ± 0.3 MF); optical density was lowest at pH 4.7 (4.5 ± 0.2 MF, *p* < *0.05*) and pH 7.3 (4.5 ± 1.0 MF, *p* < *0.05*) compared to pH 5.8 (Table 2). Glucose utilization was higher at pH 5.8 (−12.2 ± 0.2 mM, *p* < *0.01*) and pH 6.3 (−11.9 ± 0.0 mM, *p* < *0.05*) compared to pH 4.7 (2.5 ± 3.2 mM); highest metabolite production was observed at pH 6.3 (46.5 ± 1.5 mM) followed by pH 5.8 (43.1 ± 2.2 mM) and pH 6.9 (43.1 ± 4.9 mM) (Table 2). We observed a shift in fermentative activity depending on pH. Production of acetate was higher at pH 7.3 (43% of all metabolites) compared to pH 4.7 (35%, *p* < *0.05*) and pH 5.2 (22%) (Table 2). The proportion of butyrate was similar at all evaluated pH conditions (16–23%), however there was higher production at pH 5.8 (8.5 ± 0.2 mM) and pH 6.1 (8.3 ± 0.2 mM) compared to pH 4.7 (4.1 ± 0.4 mM, *p* < *0.05*). Formate was not produced at pH 4.7 or pH 7.3, and levels were highest pH 6.3 with 9.9 ± 0.9 mM (Table 2). Most lactate was produced at pH 4.7 (8 ± 1.4 mM). Levels of isovalerate (3.1–3.7 mM) were similar at all pH, while isobutyrate ranged from 1.4 mM (pH 5.5) to 7.9 mM (pH 6.3) (Table 2).

**Table 2:** Growth and metabolic activity of *C. difficile DSM* 12056 at different pH. *C. difficile* was grown in WCSP medium at pH 4–7–7.3.3, and optical density (MF) was recorded after 24 h incubation at 37 °C. Substrate utilization and metabolite formation was determined using HPLC-RI. To test statistical differences, One-way ANOVA was employed with pairwise comparison, *p* < *0.05* was considered significant. Different letters denote differences in turbidity/concentration depending on pH

pH	Optical density (MF)	Substrate consumption/metabolite formation (mM)							
		**Glucose**	**Acetate**	**Butyrate**	**Formate**	**Lactate**	**Isobutyrate**	**Isovalerate**	**Ethanol**
**4.7**	4.5 ± 0.2^a^	2.5 ± 3.2^d^	7.0 ± 0.3^a^	4.1 ± 0.4^a^	0.0 ± 0.0^a^	2.8 ± 1.4^abc^	4.1 ± 0.1^b^	3.1 ± 0.2^a^	−1.0 ± 1.1^a^
**5.2**	5.1 ± 0.4^a^	−7.3 ± 0.8^c^	6.6 ± 0.6^a^	4.8 ± 0.4^ab^	6.4 ± 0.2^bc^	1.3 ± 0.4^a^	4.9 ± 0.4^bc^	3.7 ± 0.1^ab^	2.2 ± 1.9^bcd^
**5.5**	7.5 ± 0.3^ cd^	−11.5 ± 0.3^ab^	7.6 ± 0.5^ab^	6.9 ± 0.4^ cd^	8.2 ± 0.3^bc^	2.0 ± 0.5^ab^	1.4 ± 0.6^a^	3.5 ± 0.5^ab^	0.4 ± 0.6^ab^
**5.8**	8.5 ± 0.3^d^	−12.2 ± 0.2^a^	8.5 ± 0.2^bc^	8.5 ± 0.2^e^	9.4 ± 0.2^bc^	3.9 ± 0.0^bc^	7.2 ± 1.3^d^	3.4 ± 0.5^ab^	2.2 ± 0.5^bcd^
**6.1**	7.7 ± 0.1^ cd^	−8.5 ± 0.1^bc^	7.6 ± 0.3^ab^	8.3 ± 0.4^e^	8.9 ± 0.4^bc^	2.7 ± 0.2^abc^	6.5 ± 0.0^ cd^	3.8 ± 0.1^ab^	3.3 ± 0.1^ cd^
**6.3**	6.8 ± 0.7^bc^	−11.9 ± 0.0^a^	9.8 ± 0.3^c^	7.8 ± 0.4^de^	9.9 ± 0.9^c^	4.5 ± 0.8^c^	7.9 ± 0.4^d^	3.1 ± 0.2^a^	3.6 ± 0.5^ cd^
**6.5**	6.8 ± 0.7^bc^	−10.1 ± 0.6^abc^	7.2 ± 0.5^ab^	7.5 ± 0.5^de^	11.0 ± 2.4^c^	1.7 ± 0.5^a^	5.0 ± 0.7^bc^	4.3 ± 0.4^b^	1.6 ± 0.2^abc^
**6.9**	5.6 ± 0.7^ab^	−9.4 ± 0.0^abc^	14.1 ± 0.7^d^	7.8 ± 0.2^de^	4.8 ± 4.2^b^	1.8 ± 0.5^a^	6.7 ± 0.9^ cd^	3.2 ± 0.4^a^	4.7 ± 1.1^d^
**7.3**	4.5 ± 1.0^a^	−7.4 ± 0.0^c^	15.1 ± 0.4^d^	5.7 ± 1.0^bc^	0.0 ± 0.0^a^	1.6 ± 0.8^a^	7.7 ± 1.2^d^	3.3 ± 0.5^ab^	1.6 ± 1.2^abc^

Consistent with findings made in fecal samples collected from infants of the CARE cohort, *C. difficile* responded to differences in environmental pH. Acidic pH reduced both growth and fermentation activity of *C. difficile *in vitro.

### The Antimicrobial Activity of SCCA Depended on pH

**Fig. 2:**
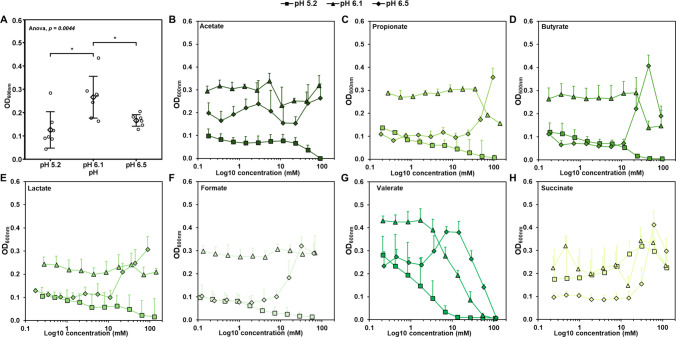
Effect of short-chain carboxylic acid (SCCA) on *C. difficile *growth at different pH. *C. difficile* DSM 12056 was grown anaerobically in WCSP in 96-well microtiter plates and antimicrobial activity of SCCA was determined two-fold broth dilution assay. The pH was adjusted to 5.2, 6.1 and 6.5. *C. difficile *was added at 10% (vol/vol) and plates were incubated anaerobically at 37 °C for 24 h. Optical density was recorded with a plate reader at 600 nm (OD_600nm_). Controls were grown without SCCA. **(A) **Optical density of *C. difficile *grown at different pH without SCCA or combined with **(B) **acetate, **(C) **propionate, **(D) **butyrate, **(E) **lactate, **(F) **formate, **(G) **valerate and **(H)** succinate. Results are average values from biological triplicates with standard deviation

After identifying the pH-dependent growth range of *C. difficile*, we assessed the antimicrobial activity of SCCA in addition to environmental (medium) pH in 96-well microtiter plates. The pH of WCSP was adjusted to pH 5.2, 6.1, and 6.5. In agreement with results in Hungate tubes*, C. difficile* grew to lower density at pH 5.2 (0.13 ± 0.08 OD_600nm_) compared to pH 6.1 (0.27 ± 0.09 OD_600nm_, *p* < *0.05*) and pH 6.5 (0.17 ± 0.02 OD_600nm_) in controls without SCCA (Fig. 2A). At pH 5.2, most of the SCCA reduced optical density in a concentration dependent manner. Accounting for the weak acid theory [19] and also under consideration of compound hydrophobicity [23], the most antimicrobial SCCA were valerate (MIC_50_ 1.6 ± 0.4 mM), butyrate (MIC_50_ 12.1 ± 6.5 mM) and propionate (MIC_50_ 5.1 ± 3.7 mM) (Fig. 2B-G). Acetate reduced optical density at the two highest concentrations, but a MIC_50_ could not be determined (Fig. 2B). The MIC_50_ of formate was 2.7 ± 1.8 mM, even though formate is a comparatively strong weak acid with low hydrophobicity. High inhibition activity was also observed in a previous study [23] possibly due to a different mode of action compared to the other SCCA. At pH 6.1 MIC_50_ could only be calculated for valerate (12.1 ± 1.2 mM) while butyrate and propionate reduced optical density at concentrations above 22 and 35 mM, respectively (Fig. 2 C, D and G). Acetate, formate and lactate did not affect optical density at pH 6.1 in agreement with the weak acid theory. At pH 6.5, optical density was generally lower than at pH 6.1 and at concentrations up to 10 mM, the presence of SCCA did not affect optical density. At levels ranging from 63 to 122 mM, all SCCA led to higher optical density (Fig. 2B-G), except for valerate. A similar pH dependent growth-supporting effect was observed for *Escherichia coli*, which grew in the presence of SCCA at pH 7.4 but was inhibited pH 6.5 [40], highlighting that the response of enteric pathogens to the presence of SCCA depends on environmental pH and concentration of SCCA. Enhanced growth in the presence of lactate could be due to the reversible activity of lactate dehydrogenase (LDH) [41]. Acetate can be used for butyrate production [42]. Compared to most SCCA, the response of *C. difficile* to succinate differed. *C. difficile* uses succinate as carbon source [6], and in agreement, succinate increased optical density when present at concentration > 14.7 mM compared to controls at pH 5.2 and 6.1, and also led to higher density at pH 6.5 (Fig. 2H). We observed that the optimal pH in vitro was lower compared to the pH with highest levels of *C. difficile*in vivo possibly due to differences in environmental conditions (e.g. presence of SCCA) or because of interactions with other intestinal microbes.

**Fig. 3:**
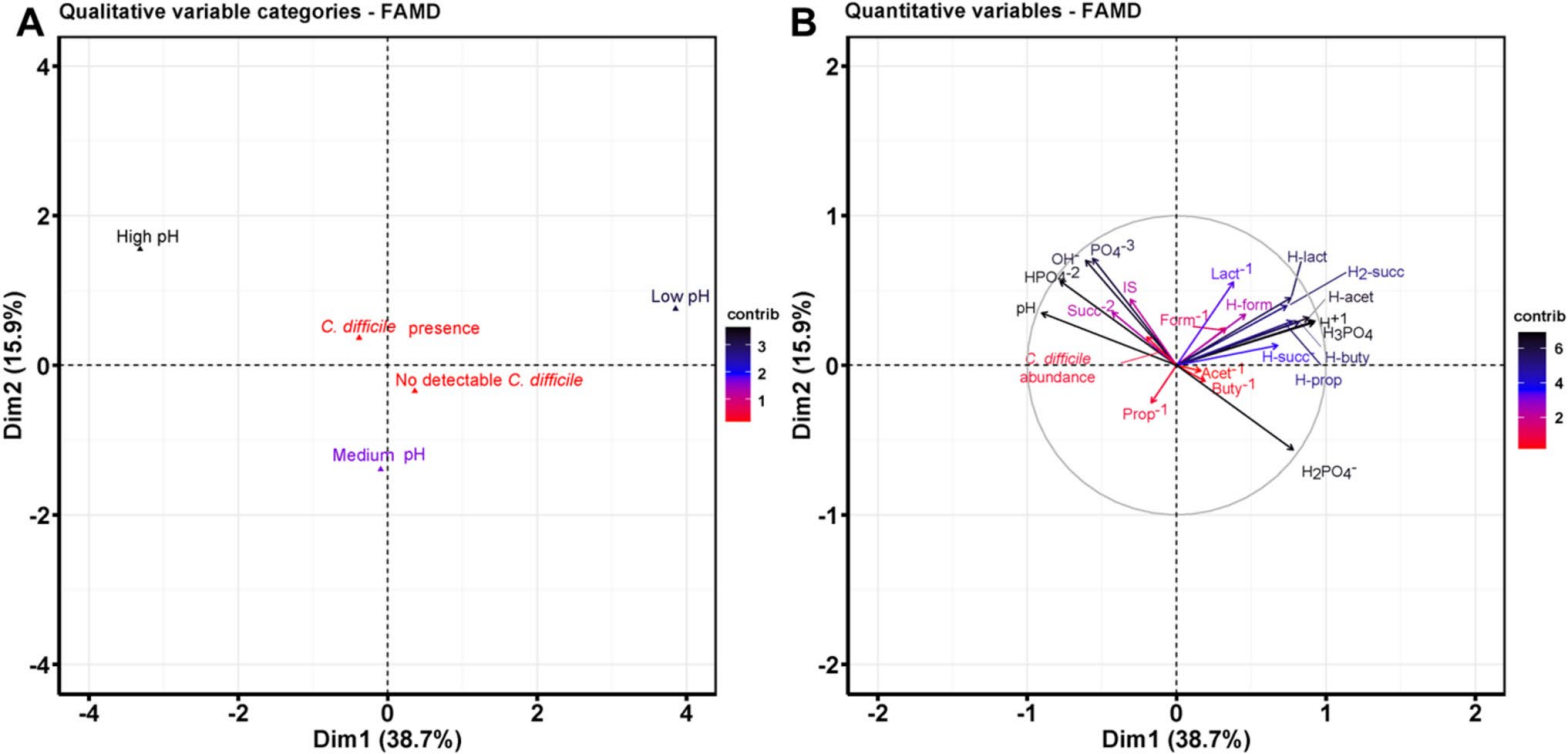
Relationship of fecal *C. difficile *occurrence, pH, SCCA levels and dissociation state. Factor Analysis of Mixed Data (FAMD) was conducted including categorical variables ‘*C. difficile* presence/absence’, and fecal pH and quantitative variables. pH categories were classified according to the quartiles (*n=35*); ‘Low pH’ (pH ≤ 5.3, *n=9*); ‘Medium pH’ (5.3 ≤ pH ≤ 6.5, *n=16*) and ‘High pH’ (pH ≥ 6.5, *n=10*). For the presence of *C. difficile* (*n=17*), absolute abundance values were used, any value above the detection limit was considered presence (> 4.9 log_10_ cells/g feces). Quantitative data included the absolute abundance of *C. difficile*, and ionic state strength and dissociation state of SCCA and PB determined using VisualMINTEQ^31^. A total of *n*=*35* fecal samples were included

Taken together, we observed that SCCA conferred antimicrobial activity in a pH- and compound dependent manner with the exception of succinate, which can be used by *C. difficile* to produce butyrate, acetate and formate [6].

### The Addition of PB Enhanced the Antimicrobial Activity of Propionate and Succinate

In the human adult gut, environmental pH is regulated by bicarbonate buffer [21] and to lesser extent phosphate buffered [20] systems. Much less is known about the intestinal buffering systems in infants, but it has been reported that serum phosphate levels are higher in infants than in adults [32], which could be an indication of a higher proportion of phosphate-based buffering. To test if the presence of a buffer system impacted the interactions of SCCA and *C. difficile*, we conducted growth experiments in the presence of PB. PB is accepted as a biorelevant buffer system in studies simulating the small intestine [43–45]. We utilized the pH ranges employed on the microtiter plates and also tested at pH 5.8, which allowed for best growth in Hungate tubes.

The effect of PB on optical density was pH-dependent (Table 3), with no clear impact of increasing PB concentrations (Table 3). At pH 5.2, optical density in the presence PB of was generally low (range 0.8–2.4 MF) and optical density was similar to controls without PB at pH 5.6–5.8 and pH 6.1 (Table 3). At pH 5.6–5.8, 6.1 and 6.5, *C. difficile* used succinate and produced mainly acetate, butyrate and formate (Table 3) [6]. In accordance with observations made in microtiter plates, the addition of succinate did not affect final optical density while propionate reduced growth compared to controls (Table 3). When added in combination, PB (100 mM) and both SCCA reduced (*p* < *0.05*) optical density at pH 5.6–5.8 and pH 6.1 (Table 3).

**Table 3:** Growth and metabolic activity of *C. difficile DSM* 12056 at different pH with SCCA and phosphate buffer. *C. difficile* was grown in WCSP medium at pH 5.2, 5.8, 6.1, 6.5 that was supplied with PB (10, 50 or 100 mM), succinate (100-110 mM) or propionate (100-150 mM) also in combination. Turbidity was recorded after 24 h incubation at 37 °C. Substrate utilization and metabolite formation was determined using HPLC-RI. To test for statistical differences, One-way ANOVA was employed with pairwise comparison using Tukey test, p < 0.05 was considered significant. Capital letters denote differences in turbidity/concentration within the same pH across different conditions. Small letters denote differences in turbidity/concentration at the same condition depending on pH

pH	Treatment	Optical density	Substrate utilization and metabolite formation (mM)									
		(MF units)	Glucose	Acetate	Butyrate	Formate	Lactate	Propionate	Isobutyrate	Isovalerate	Ethanol	Succinate
5.2	Control	4.1 ± 0.1^B, a^	−2.8 ± 0.3^BC, b^	5.0 ± 0.2^CDE, a^	4.2 ± 0.1^B, a^	6.5 ± 0.4^C, a^	1.0 ± 0.7^C, a^	0.0 ± 0.0^BC, a^	4.9 ± 0.2^C, a^	3.3 ± 0.2^E, a^	0.0 ± 0.0^B, a^	0.0 ± 0.0^B, b^
	PB10	2.4 ± 0.5^AB, a^	−5.7 ± 3.6^A, b^	3.4 ± 0.4^BCDE, a^	3.3 ± 0.3^B, a^	0.0 ± 0.0^BC, a^	−1.0 ± 1.5^BC, a^	0.0 ± 0.0^BC, a^	0.0 ± 0.0^BC, a^	2.1 ± 1.9^DE, a^	0.0 ± 0.0^B, a^	0.0 ± 0.0^B, b^
	PB50	2.3 ± 0.7^AB, a^	−3.2 ± 0.3^BC, b^	4.1 ± 0.7^E, a^	3.2 ± 0.3^B, a^	6.0 ± 0.3^C, a^	−0.1 ± 0.3^C, a^	0.0 ± 0.0^BC, a^	0.0 ± 0.0^C, a^	2.6 ± 0.1^E, a^	0.0 ± 0.0^B, a^	0.0 ± 0.0^B, b^
	PB100	0.8 ± 0.3^A, a^	0.3 ± 0.2^C, b^	1.9 ± 0.9^DE, a^	0.7 ± 1.3^B, a^	0.0 ± 0.0^C, a^	0.1 ± 0.5^C, a^	2.6 ± 4.5^C, a^	−1.6 ± 2.7^C, a^	0.0 ± 0.0^CDE, a^	0.0 ± 0.0^B, a^	0.0 ± 1.4^B, b^
	Succ-PB100	0.0 ± 0.1^A, a^	0.4 ± 0.7D^, b^	0.3 ± 0.3^AB, a^	0.0 ± 0.0^A, a^	0.0 ± 0.0^AB, a^	0.0 ± 0.0^AB, a^	2.1 ± 4.3^C, a^	0.0 ± 0.0^A, a^	0.0 ± 0.0^AB, a^	0.0 ± 0.0^AB, a^	3.9 ± 5.4^B, b^
	Prop	0.1 ± 0.1^A, a^	−2.7 ± 0.6^C, b^	0.8 ± 0.2^ABCD, a^	2.3 ± 2.0^A, a^	0.0 ± 0.0^BC, a^	−0.8 ± 0.9^AB, a^	−10.5 ± 7.3^B, a^	0.0 ± 0.0^A, a^	0.0 ± 0.0^ABC, a^	0.0 ± 0.0^AB, a^	0.0 ± 0.0^B, b^
	Prop-PB10	−0.1 ± 0.1^AB, a^	−2.0 ± 0.2^C, b^	0.6 ± 0.2^BCDE, a^	1.8 ± 1.6^A, a^	0.0 ± 0.0^ABC, a^	−0.5 ± 0.8^AB, a^	−9.1 ± 3.6^B, a^	0.0 ± 0.0^AB, a^	0.7 ± 1.3^BCD, a^	0.0 ± 0.0^AB, a^	0.0 ± 0.0^B, b^
	Prop-PB50	−0.1 ± 0.0^A, a^	−5.2 ± 3.8^BC, b^	0.0 ± 0.3^ABC, a^	0.0 ± 0.0^A, a^	0.0 ± 0.0^ABC, a^	−1.4 ± 0.8^A, a^	−35.5 ± 34.8^A, a^	0.0 ± 0.0^A, a^	0.0 ± 0.0^ABC, a^	0.0 ± 0.0^AB, a^	0.0 ± 0.0^B, b^
	Prop-PB100	−0.1 ± 0.0^A, a^	0.3 ± 0.6^D, b^	0.1 ± 0.1^A, a^	0.0 ± 0.0^A, a^	0.0 ± 0.0^A, a^	0.0 ± 0.8^AB, a^	3.3 ± 7.4^BC, a^	0.0 ± 0.0^A, a^	0.0 ± 0.0^A, a^	0.0 ± 0.0^B, a^	0.1 ± 1.3^B, b^
5.6–5.8	Control	6.0 ± 0.3^CD, ab^	−4.7 ± 0.6^BC, b^	6.4 ± 0.1^CDE, c^	6.1 ± 0.5^C, b^	7.5 ± 0.2^C, b^	2.2 ± 0.6C, b	0.0 ± 0.0^BC, a^	6.5 ± 0.3^C, b^	4.7 ± 0.2^E, b^	0.0 ± 0.0^B, ab^	0.0 ± 0.0^B, b^
	PB10	4.9 ± 0.4^CD, b^	−6.9 ± 2.5A^, b^	4.5 ± 0.1^BCDE, b^	5.2 ± 0.3^B, b^	7.3 ± 0.1^BC, b^	0.7 ± 1.1^BC, b^	0.0 ± 0.0^BC, a^	5.4 ± 0.4^BC, b^	3.9 ± 0.5^DE, b^	0.0 ± 0.0^B, ab^	0.0 ± 0.0^B, b^
	PB50	5.2 ± 0.4^CD, b^	−5.2 ± 0.9^BC, b^	5.8 ± 0.1^E, b^	5.5 ± 0.1^b, b^	7.6 ± 0.3^C, b^	1.5 ± 0.1^C, b^	0.0 ± 0.0^BC, a^	8.1 ± 1.8^C, b^	4.4 ± 0.5^E, b^	0.8 ± 1.4^B, ab^	0.0 ± 0.0^B, b^
	PB100	7.1 ± 0.1^D, b^	−3.0 ± 0.6^C, b^	6.9 ± 0.3^DE, b^	7.1 ± 0.2^B, b^	8.5 ± 0.1^C, b^	2.6 ± 0.1^C, b^	2.4 ± 3.6^C, a^	7.0 ± 1.8^C, b^	3.5 ± 0.7^CDE, b^	0.0 ± 0.0^B, ab^	−0.2 ± 1.1^B, b^
	Succ	7.1 ± 0.3^D, a^	−6.8 ± 0.1A^B, b^	14.5 ± 0.6^F, b^	9.0 ± 1.1^A, b^	7.4 ± 0.5^BC, b^	0.0 ± 0.0^AB, b^	0.0 ± 0.0^BC, b^	6.6 ± 0.7^C, b^	4.3 ± 0.5^DE, b^	0.0 ± 0.0^A, ab^	−13.9 ± 0.7^A, b^
	Succ-PB100	1.3 ± 0.4^AB, ab^	−0.2 ± 0.2^D, b^	3.1 ± 1.1^AB, b^	2.9 ± 0.3^A, b^	0.4 ± 0.7^AB, b^	0.0 ± 0.0^AB, b^	1.9 ± 3.6^C, a^	0.0 ± 0.0^A, b^	0.8 ± 1.3^AB, b^	0.0 ± 0.0^AB, ab^	3.8 ± 6.4^B, b^
	Prop	1.2 ± 0.7^AB, ab^	−3.9 ± 0.7^C, b^	2.7 ± 0.8^ABCD,b^	3.3 ± 0.4^B, b^	4.1 ± 3.6^BC, b^	−0.6 ± 0.6^AB, b^	−11.1 ± 5.3^B, a^	0.0 ± 0.0^A, b^	0.9 ± 1.6^ABC, b^	0.0 ± 0.0^AB, ab^	0.0 ± 0.0^B, b^
	Prop-PB10	1.2 ± 0.2^BC, ab^	−3.1 ± 0.2^C, b^	2.6 ± 0.2^BCDE, b^	2.2 ± 1.9^A, b^	4.3 ± 3.7^ABC, b^	0.3 ± 0.1^AB, b^	−10.2 ± 0.5^B, a^	0.0 ± 0.0^AB, a^	1.8 ± 1.6^BCD, b^	0.0 ± 0.0^AB, ab^	0.0 ± 0.0^B, b^
	Prop-PB50	1.6 ± 0.5^AB, ab^	−5.6 ± 1.3^BC, b^	3.0 ± 0.4^ABC, b^	3.2 ± 0.3^A, b^	4.3 ± 3.8^ABC, b^	−1.1 ± 1.4^A, b^	−32.2 ± 7.7^A, a^	0.0 ± 0.0^A, b^	0.0 ± 0.0^ABC, b^	0.0 ± 0.0^AB, ab^	0.0 ± 0.0^B, b^
	Prop-PB100	0.1 ± 0.2^A, a^	0.1 ± 0.4^D, b^	0.6 ± 0.1^A, a^	1.0 ± 1.8^A, b^	0.0 ± 0.1^A, b^	0.0 ± 0.3^AB, b^	1.0 ± 4.6^BC, a^	0.0 ± 0.0^A, b^	0.0 ± 0.0^A, b^	0.8 ± 1.4^B, ab^	0.0 ± 1.3^B, b^
6.1	Control	7.7 ± 0.1^CD, b^	−8.5 ± 0.1^BC, a^	7.6 ± 0.3^CDE, c^	8.3 ± 0.4^C, c^	8.9 ± 0.4^C, c^	2.7 ± 0.2^C, b^	0.0 ± 0.0^BC, a^	6.5 ± 0.0^C, bc^	3.8 ± 0.1^E, b^	3.3 ± 0.1^B, b^	0.0 ± 0.0^B, b^
	PB10	8.4 ± 0.2^CD, c^	−14.3 ± 2.4^A, a^	7.4 ± 0.1^BCDE, c^	9.0 ± 0.5^B, c^	8.7 ± 0.9^BC, c^	2.0 ± 1.4^BC, b^	0.0 ± 0.0^BC, a^	6.8 ± 0.4^BC, bc^	4.1 ± 0.6^DE, b^	3.4 ± 3.1^B, b^	0.0 ± 0.0^B, b^
	PB50	7.5 ± 0.3^CD, b^	−8.4 ± 0.1^BC, a^	7.6 ± 0.3^E, c^	8.4 ± 0.5^B, c^	9.0 ± 0.9^C, c^	2.7 ± 0.6^C, b^	2.3 ± 3.9^BC, a^	6.7 ± 0.2^C, bc^	3.7 ± 0.5^E, b^	3.7 ± 0.5^B, b^	0.0 ± 0.0^B, b^
	PB100	8.1 ± 0.2^CD, b^	−6.4 ± 0.6^C, a^	8.2 ± 0.5^DE, c^	9.2 ± 0.2^B, c^	10.4 ± 0.4^C, c^	3.2 ± 0.4^C, b^	4.4 ± 3.8^C, a^	6.5 ± 1.6^C, bc^	3.5 ± 0.4^CDE, b^	1.2 ± 2.1^B, b^	0.0 ± 1.2^B, b^
	Succ	8.9 ± 0.5^D, a^	−10.4 ± 1.2^AB, a^	21.7 ± 2.0^F, c^	15.1 ± 1.2^A, c^	10.1 ± 1.1^BC, c^	0.0 ± 0.0^AB, b^	0.0 ± 0.0^BC, a^	7.1 ± 1.2^C, bc^	3.7 ± 0.2DE, b	−1.8 ± 0.8^A, b^	−12.6 ± 7.3^A, b^
	Succ-PB100	2.5 ± 0.3^AB, ab^	0.1 ± 0.2^D, a^	4.5 ± 1.2^AB, c^	3.7 ± 0.8^A, c^	6.6 ± 5.7^AB, c^	0.0 ± 0.0^AB, b^	0.7 ± 0.9^C, a^	1.9 ± 3.3^A, bc^	1.0 ± 1.7^AB, b^	−0.2 ± 0.3^AB, b^	1.5 ± 5.3^B, b^
	Prop	2.9 ± 0.5^AB, bc^	−4.4 ± 0.3^C, a^	5.8 ± 0.3^ABCD, c^	3.4 ± 0.5^B, c^	6.9 ± 1.3^BC, c^	−0.7 ± 0.9^AB, b^	−12.0 ± 6.4^B, a^	0.0 ± 0.0^A, bc^	2.1 ± 1.8^ABC, b^	0.0 ± 0.0^AB, b^	0.0 ± 0.0^B, b^
	Prop-PB10	4.2 ± 0.1^BC, b^	−4.8 ± 0.^C, a^	8.7 ± 0.7^BCDE, c^	4.2 ± 0.4^A, c^	7.5 ± 0.5^ABC, c^	−0.2 ± 0.5^AB, b^	−10.4 ± 0.2^B, a^	5.0 ± 0.4^AB, bc^	3.0 ± 0.2^BCD, b^	0.0 ± 0.0^AB, b^	0.0 ± 0.0^B, b^
	Prop-PB50	2.8 ± 0.4^AB, b^	−4.7 ± 0.9^BC, a^	5.3 ± 0.7^ABC, c^	3.8 ± 0.3^A, c^	6.9 ± 0.8^ABC, c^	−0.4 ± 0.9^A, b^	−10.2 ± 9.7^A, a^	0.0 ± 0.0^A, bc^	2.9 ± 0.4^ABC, b^	0.0 ± 0.0^AB, b^	0.0 ± 0.0^B, b^
	Prop-PB100	0.1 ± 0.1^A, a^	0.0 ± 0.6^D, a^	0.3 ± 0.1^A, c^	0.9 ± 1.6^A, c^	0.1 ± 0.1^A, c^	0.0 ± 0.1^AB, b^	1.1 ± 1.1^BC, a^	0.0 ± 0.0^A, bc^	0.0 ± 0.0^A, b^	0.4 ± 0.7^B, b^	0.5 ± 0.8^B, b^
6.5	Control	6.8 ± 0.7^B, ab^	−9.5 ± 0.6^BC, a^	7.2 ± 0.5^CDE, d^	7.5 ± 0.5^C, c^	9.5 ± 2.5^C, c^	1.0 ± 1.7^C, b^	−0.1 ± 0.9^BC, a^	5.0 ± 0.7^C, c^	4.3 ± 0.4^E, c^	0.0 ± 0.0^B, b^	−0.7 ± 0.7^B, a^
	PB100	3.0 ± 0.2^A, a^	−6.9 ± 2.4^C, a^	9.5 ± 1.3^DE, d^	7.1 ± 0.2^B, c^	12.7 ± 0.2^C, c^	1.7 ± 0.7^C, b^	−1.4 ± 1.2^C, a^	8.5 ± 0.9^C, c^	4.0 ± 0.1^CDE, c^	5.0 ± 1.4^B, b^	0.0 ± 0.0^B, a^
	Succ	6.7 ± 1.2^B, a^	−10.0 ± 0.1^AB, b^	24.3 ± 0.5^F, d^	14.6 ± 0.9^A, c^	6.4 ± 6.4^BC, c^	0.0 ± 0.0^AB, b^	0.7 ± 0.5^BC, a^	7.1 ± 1.3^C, c^	3.4 ± 0.6^DE, c^	−2.6 ± 0.6^A, b^	−21.3 ± 1.6^A, a^
	Succ-PB100	3.3 ± 0.4^A, b^	−2.0 ± 3.7^D, a^	8.8 ± 0.9^AB, d^	4.8 ± 0.8^A, c^	8.3 ± 4.5^AB, c^	0.0 ± 0.0^AB, b^	−0.7 ± 0.7^C, a^	4.4 ± 0.3^A, c^	3.6 ± 0.2^AB, c^	0.8 ± 4.8^AB, b^	−15.5 ± 18.9^B,a^
	Prop	4.7 ± 0.8^AB, c^	−6.7 ± 0.8^C, a^	9.4 ± 0.4^ABCD, d^	5.5 ± 0.3^B, c^	12.1 ± 1.0^BC**,** c^	1.7 ± 0.5^AB, b^	−6.0 ± 4.4^B, a^	0.0 ± 0.0^A, c^	3.4 ± 0.4^ABC, c^	0.0 ± 0.0^AB, b^	0.1 ± 0.1^B, a^
	Prop-PB100	4.7 ± 1.0^AB, b^	−3.0 ± 3.1^D, a^	10.1 ± 0.5^A, d^	5.3 ± 0.2^A, c^	6.9 ± 4.0^A, c^	0.0 ± 0.9^AB, b^	−12.9 ± 14.1^BC, a^	5.2 ± 1.2^A, c^	3.2 ± 0.2^A, c^	1.9 ± 2.0^B, b^	0.0 ± 0.0^B, a^

The presence of PB (100 mM) prevented succinate utilization and fermentation activity at pH 5.6–5.8, 6.1 (*p* < *0.05*) and decreased uptake of succinate by 69% at pH 6.5 (Table 3). Similarly, there was little or no propionate uptake and fermentation activity when *C. difficile* was grown with 100 mM PB and propionate (Table 3). In contrast, > 40 mM propionate was depleted from the medium when *C. difficile* was grown in the presence of PB (50 mM*, p* < *0.05*) at pH 6.1 and 5.6–5.8 with no or low detectable formation of fermentation metabolites (Table 3).

Taken together, these results suggested a synergistic antimicrobial effect of 100 mM PB with succinate and propionate reducing growth and SCCA in a pH dependent manner. *C. difficile* possesses a succinate specific transporter [6], while propionate is passing through the bacterial membrane via diffusion. Surprisingly, phosphate buffer increased the intracellular levels of propionate when added at 50 mM at 5.2 and 5–6-5.8.8 suggesting that interactions of PB with the membrane led to higher diffusion. In previous work, the presence of a bicarbonate buffer system increased susceptibility to antibiotics due to changes of the transmembrane proton motive force [46].

### MLR Analysis Identified Statistical Interactions of PB and SCCA

To statistically validate our observation of synergistic activity of PB and SCCA, we conducted MLR analysis. We used optical density data recorded when *C. difficile* was grown at different pH, in the presence of PB and/or propionate, succinate. We conducted and included additional experiments of growth with other SCCA (Suppl. Table S1 and Table S3A) and tested how optical density was affected by pH and interactions between SCCA and PB (Eq. 1). MLR model 1 explained 40% of the variation in optical density. pH had a positive effect (*p* < *0.001*) on optical density. PB only affected optical density negatively (*p* < *0.05*) in combination with the presence of butyrate, lactate, propionate, succinate and valerate. These results support the synergistic antimicrobial effect of PB and specific SCCA observed in our growth experiments.

### Ionic Strength Contributed to the Growth Reduction

Additionally, we determined the individual contribution of SCCA on optical density using another MLR model 2. Beside pH, we included ionic strength as an explanatory variable in MLR model 2, since both SCCA and PB are weak acids. In general, ionic strength increased with increasing pH and was around twofold higher in the presence of PB and/or SCCA (Suppl. Table S2 and Table S3). The MLR model 2 explained 63% of the variation in optical density. Both pH and ionic strength had an effect (*p* < *0.01*) on optical density. Ionic strength was negatively related (estimate = −18.8) while pH was positively associated with optical density (estimate = 3.4). The presence of SCCA butyrate (estimate = −3.9), lactate (estimate = −3.3), propionate (estimate = −2.9) and valerate (estimate = −4.4) negatively affected (*p* < *0.001*) optical density. The MLR model 2 identified valerate as the strongest antimicrobial SCCA, in agreement with results obtained in this study (Suppl. Table S3). Our data highlights the importance of accounting for ionic strength when evaluating the potential antimicrobial effect of SCCA, PB and pH.

### In vivo*, *the presence of *C. difficile *Inversely Related to Hydrated SCCA and Buffer Compounds

After identifying the relationship of pH, ionic strength and the presence of SCCA on growth *C. difficile *in vitro, we determined how pH and the dissociation state of SCCA related to the presence or absence of *C. difficile *in vivo again employing again data collected from the CARE cohort. We estimated the dissociation state of SCCA present in feces using VisualMINTEQ [31] (Suppl. Table S3).

At the pH prevailing in fecal samples, acetate was the most prevalent form of the SCCA (83.6, IQR 71.7‒104.3 µmol/g feces) followed by butyrate (11.5, IQR 5.5‒18.3 µmol/g feces). The most prevalent ion of the PB system was H_2_PO_4_^−^ (18.6, IQR 14.1‒19.4 mM), based on an estimated concentration of 20 mM in the gut lumen (Suppl. Table S3).

We employed multivariate FAMD analysis to further investigate the relationship of *C. difficile* occurrence and abundance and the prevalence of SCCA in a pH-dependent state of dissociation in vivo (Suppl. Table S3). In the FAMD analysis, the 1 st and 2nd dimension explained 38.7% and 15.9%, respectively (Fig. 3). The qualitative variables ‘High pH’ and ‘*C. difficile* presence’ were located in the same quadrant (Fig. 3A). Based on quantitative variables, ‘*C. difficile* abundance’ was related to ionic strength (‘IS’), and the most dissociated states of PB such as ‘PO_4_^−3^’, ‘HPO_4_^−2^’, and SCCA (‘Succ-2’), while they were inversely related to the undissociated SCCA (‘H-acet’, ‘H2-succ’, ‘H-lact’, ‘H-form’, ‘H-buty’ and ‘H-prop’) (Fig. 3B). Moreover, ‘*C. difficile* abundance’ was inversely related to ‘H_2_PO^4−^’. Taken together, our data links occurrence of *C. difficile* to pH and chemical state of SCCA and a buffer system.

## Conclusion

The gastrointestinal tract hosts a complex ecosystem shared by microbes and metabolites formed by fermentation activity from dietary components. With this study, we shed light on how chemical systems (SCCA, pH, buffer systems, ionic strength) affect performance of the enteropathogen *C. difficile.* Our findings reveal that environmental pH plays a crucial role on growth and fermentative activity of *C. difficile*. The impact of SCCA was compound-, concentration- and pH-dependent with inhibitory activity at pH 5.2 and 6.1, and growth promotion at pH 6.5. The presence of PB had an apparent combined antimicrobial effect with SCCA at low pH, which may contribute to low colonization of *C. difficile *in vivo. Our findings show that the dissociation state of SCCA and PB, which relates to ionic strength, is an important factor that contributes to the interactions of SCCA and *C. difficile*.

## Supplementary Information

Below is the link to the electronic supplementary material.

Supplementary file1 (XLSX 215 KB)

## Data Availability

We retrieved the 16S rRNA gene dataset generated by Appert et al. from ENA (PRJNA616703) and processed the data as described.
